# Optimizing Mucoadhesive Film-Forming Spray for Efficient Oral Delivery of Fluconazole in Candidiasis Treatment

**DOI:** 10.7759/cureus.70359

**Published:** 2024-09-27

**Authors:** Ilaf J Atoosh, Mowafaq M Ghareeb

**Affiliations:** 1 Department of Pharmacy, Ministry of Health and Environment, Baghdad, IRQ; 2 Department of Pharmaceutics, College of Pharmacy, University of Baghdad, Baghdad, IRQ

**Keywords:** fluconazole, hyaluronic acid, mucoadhesive film forming spray, oral candidiasis, polyvinyl alcohol

## Abstract

Buccal candidiasis has become increasingly prevalent in recent years, with *Candida albicans* being the primary causative organism. While systemic fluconazole is an effective treatment, its use is associated with adverse effects such as gastric upset, hepatic failure, and potential drug interactions. Therefore, the development of local fluconazole treatment presents a promising solution to these challenges. This study aimed to formulate an efficient local mucoadhesive film-forming spray for the targeted delivery of fluconazole in the treatment of oral candidiasis. The investigation involved the use of three polymers (hyaluronic acid, polyvinyl alcohol, and xanthan gum) both individually and in combination to identify the most effective formulation. Various tests were conducted to characterize 13 formulations prepared using these polymers, including UV-vis spectroscopy, Fourier-transform infrared spectroscopy (FTIR), content assay, drying time and film formation, viscosity determination, determination of the mucoadhesion strength by turbidimetric methods, drug release study, in-vitro anticandida activity, histological irritation analysis, and stability study. The optimum formula F11, comprised polyvinyl alcohol for its superior mechanical properties and film-forming capabilities, hyaluronic acid, and xanthan gum in combination, exhibiting synergistic mucoadhesive strength. This optimal formulation demonstrated maximum mucoadhesion, rapid film formation, an acceptable spray angle, and controlled release. Furthermore, the optimum formula underwent additional evaluations for in vitro anti-Candida activity, in vivo irritancy assessments, and stability studies, all of which yielded satisfactory results. These findings support the potential of the optimum formula as a straightforward and efficient spray formulation for the treatment of oral candidiasis.

## Introduction

An overabundance of the yeast *Candida albicans* is the main cause of Candida infections in humans [1]. Candidiasis is usually seen in mucocutaneous membranes including the oral cavity. Oral candidiasis is superficial on the mucous membrane of the oral cavity. Clinical indicators are thick, white elevated pseudomembranous. This condition is particularly common in individuals with compromised immune systems, such as those with acquired immunodeficiency syndrome (AIDS), diabetes, or those undergoing chemotherapy [2].

Candida can be treated with topical or systemic antifungals; systemic fluconazole is very effective and considered the most common drug used to treat advanced infections. When compared to other anti-fungal medications, fluconazole is more effective in lowering the resistance of candida in oral candidiasis. Fluconazole when taken systemically has side effects like nausea, diarrhea, and hepatotoxicity [3], for the treatment of oral candidiasis, it is advisable to choose local therapy as the first option to minimize side effects, prevent medication resistance, and ensure targeted delivery. The marketed buccal antifungal Clotrimazole is available as lozenges, but Clotrimazole has a narrower spectrum of activity compared to fluconazole. It may not be as effective against certain types of fungi that fluconazole can target. In addition to that, Clotrimazole lozenges often need to be used multiple times a day, which can be less convenient and impact patient compliance compared to once-daily fluconazole.

Local mucoadhesive dosage forms maintain contact with the infected area for a desirable duration with longer residence time. Oral mucoadhesive dosage forms have the advantages of being accessible, easy to use, and high patient compliance. More important these dosage forms are used for target drug delivery at the mucosa, and to reduce systemic exposure [4].

There are a lot of advantages to utilizing a spray instead of other topical dosage forms. Some of these include sterilization, simple application, low irritation risk, providing great coverage, dispersing the drug uniformly, and having a flexible dose [5]. Researchers have created effective and successful spray preparations in recent years for use in medical applications, such as film-forming sprays (FFSs) matrices. An FFS is a type of topical formulation designed to create a protective, thin layer or film over a surface, typically the skin or mucous membranes. Using by using polymers as a film matrix [6]. Dose adjustments are established based on the quantity of solution in each actuation to control both systemic and local effects. It is also easy to use as it spreads easily and uniformly.

Hyaluronic acid (HA), a natural polymer, is utilized in film systems due to its excellent biocompatibility, minimal immunogenicity [7], and strong mucoadhesion strength. The addition of film-forming polyvinyl alcohol (PVA) which is a synthetic water-soluble polymer with good mechanical properties can reduce the degradation rate of natural polymers and avoid their rapid dissolution in biological fluids [8,9]. The combination of HA and xanthan gum (XG) exhibits greater mucoadhesive strength and adhesion duration compared to each polymer alone [10]. The aim of this work is to study the variables affecting buccal mucoadhesive FFS formulation and optimize it to the best acceptable one.

## Materials and methods

Materials

Fluconazole was obtained from Meryer (China). Mucin powder type II was sourced from Shanghai-Hai D and B Biological and Technology (China), PVA 1788 was provided by Shanghai Macklin Biochemical Co., Ltd (China), HA was acquired from Beijing Zhong Shuo Pharmaceutical Technology Development Co., Ltd (China), polyethylene glycol 400, propylene glycol, and ethanol were supplied by Thomas Baker.

Methods

Characterization of Fluconazole of Fluconazole λ Max in Ethanol and PBS of 6.8

Stock solutions were made by dissolving 15 mg of fluconazole in 100 mL of each media ethanol and phosphate buffer of pH 6.8. The resulting concentration of the stock solution is 0.15 mg/mL. Using UV-vis spectrophotometry (Shimadzu, Japan), diluted solutions were produced from the stock solutions and studied at wavelengths ranging from 200 to 400 nm. The purpose was to identify the maximum absorption of fluconazole (λ max) in each medium.

Determination of Fluconazole Calibration Curves in Ethanol and PBS pH 6.8

Fluconazole calibration curves were measured in ethanol and phosphate buffer of pH 6.8. The initial step involved creating stock solutions with a concentration of 0.15 mg/mL of fluconazole in either media, from which further dilutions at different concentrations were made. The absorbance of the successive dilutions was then measured triplicate using UV-vis spectrophotometry at λ max. They were plotting the resultant absorbance against the yielded fluconazole calibration curves.

Fourier-Transform Infrared Spectroscopy (FTIR) Compatibility Test of Drugs and Excipients

The FTIR spectra of pure drug, PVA, HA, XG, and a physical mixture of pure drug and the polymers were separately analyzed using FTIR spectroscopy (Lambda 7600, USA). An analysis is done by grinding the substance with potassium bromide (Merck Life Science, Indonesia) and subsequently compacting it into a thin film disc using a precise technique. The achieved outcomes were observed within the wave number range of 4000-400 cm^-1^.

Preparation of Mucoadhesive Oral Film-Forming Solutions of Fluconazole

As shown in Table 1, the mucoadhesive polymers that were used were PVA, HA, and xanthan gum (XG). The film-forming solutions differ from one another in terms of the type of polymer used, polymer combinations, and polyethylene glycol (PEG) 400 concentrations as film-forming polymer. To prepare polymer solutions a magnetic stirrer (Joanlab, China) was used. XG was stirred with water at 80°C for 10 min, then at 25°C for two hours to complete hydration; PVA was stirred for 45 min at room temperature, and HA was stirred until a clear solution was formed. Then a fixed amount of 5% propylene glycol (PG) was added to each polymeric solution as it was used as a stabilizer of the polymer solution, plasticizer, and permeation enhancer. PG is used in this concentration because it significantly affects film-forming solution viscosity [11], and drug penetration improves with PG concentrations below 5% [12]. Both PG and PEG400 increase penetration of antifungal drugs as they solubilize and carry the drug. Fluconazole 1% w/v was dissolved in ethanol which is used as a solvent for fluconazole, and it is typically used as solvent for FFS dosage forms, it is the last was added gradually to each polymeric solution [13].

**Table 1:** Combinations and percentage of ingredients used in the preparation of film-forming solutions

Formula	Fluconazole w/v	Propylene glycol v/v	Polyvinyl alcohol w/v	Hyaluronic acid w/v	Xanthan gum w/v	Polyethylene glycol 400 v/v	Ethanol v/v	Water up to mL
F1	1	5	0.2	---	---	10	40	100
F2	1	5	0.3	---	---	10	40	100
F3	1	5	---	0.2	---	10	40	100
F4	1	5	---	0.3	---	10	40	100
F5	1	5	---	---	0.2	10	40	100
F6	1	5	---	---	0.3	10	40	100
F7	1	5	0.2	0.2	---	10	40	100
F8	1	5	0.2	0.3	---	10	40	100
F9	1	5	0.3	0.2	---	10	40	100
F10	1	5	0.3	0.3	---	10	40	100
F11	1	5	0.3	0.3	0.1	10	40	100
F12	1	5	0.3	0.3	0.1	20	40	100
F13	1	5	0.3	0.3	0.1	30	40	100

Fluconazole Content Determination in Prepared Formulas

A one-milliliter sample from each film-forming solution prepared in Table 1, which was supposed to contain 10 milligrams of FLC, was combined with 50 milliliters of ethanol. The absorbance of the produced solution was quantified by using a UV-vis spectrophotometer (Shimadzu) at lambda max 261.

Time for Drying and Film Formation

Film forming solution was sprayed on a glass petri dish, and the time required for solvent evaporation and film formation was recorded [14].

Viscosity Determination

The viscosity of each of the film-forming solutions was measured using a digital viscometer (Brookfield, USA). Measurements of viscosity were taken at 12, 30, 60, and 100 rpm, and that was done three times.

Determination of the Mucoadhesion Strength by Turbidimetric Method

First 0.1% mucin dispersion was prepared, then combinations of formulas and mucin were examined using turbidimetric measurements and compared them to the dispersion of the only mucin turbidity at a wavelength of 650 nm using an ultraviolet-visible spectrophotometer. A 5 mL sample of each formula was combined with a 5 mL mixture of (0.1%) mucin in water. The mixture was then stirred by a magnetic stirrer at a speed of 200 rpm at a temperature of 37°C. The turbidity of the dispersions was measured at 30-minute intervals using UV-vis spectrophotometry (Shimadzu) and compared to the turbidity of the only mucin dispersion. The turbidity increases of the mucin-formula demonstrated its mucoadhesion strength [15].

Drug Release Study

An in vitro release study by using a USP type II dissolution test (Faithful, China). About 1 mL of each of the F10, F11, F12, and F13 solutions was sprayed on a petri dish (by spraying the solution over a Petri dish, we can ensure the formation of a uniform film over a flat surface). After solvent ethanol evaporation and film formation, the petri dish was added to a dissolution jar with 500 mL phosphate buffer solution of pH 6.8 and mixed at 50 rpm ensuring the sink condition. The fluconazole content was determined by taking samples at certain intervals and analyzing them using a UV spectrophotometer. These formulas were chosen to study the effect of adding XG and the effect of different amounts of PEG400 on drug release.

Selection of the Optimum Mucoadhesive FFS Solution

Further study was conducted on the best formula that met the following criteria: higher mucoadhesivity, short drying time, appropriate viscosity, acceptable spray angle and drug release, also the drug content within the permitted official limit.

Agar Well Diffusion Assay for In-Vitro Anticandida Activity

The optimum preparation F11 was tested in vitro for anti-candida activity by the Muller-Hinton agar diffusion disc technique. *C. albicans* cultures were modified to 1.5×10^8^ through comparison with 0.5 McFarland. The inoculation media was used to drill wells 6 mm in diameter, and F11 and a control formula (the same ingredients of F11 without fluconazole) were carefully inserted into these wells. The plate was then incubated at 37°C. A day later, the inhibitory zone diameter was assessed.

Animal test: histological irritation study of optimum formula (F11)

Animal Selection and Preparation

Six male rats were selected for the study. The rats underwent a fasting period of at least one hour prior to the initiation of the experiment to ensure consistency in food intake. Throughout the study, the rats were deprived of food but were allowed access to water 60 minutes after the oral application of the liquid formula.

Application and Observation

The buccal mucosa of the rats was exposed to the oral liquid solution (F11) using an art brush for a duration of 30 minutes. Macroscopic examinations were conducted before application and at one, two, and 24 hours post-application on each animal to assess for signs of ulcers, hemorrhaging, color changes, or sloughing, following the safety evaluation guidelines of the Cosmetic, Toiletry, and Fragrance Association [16].

Euthanasia and Tissue Processing

After 24 hours, the rats were humanely euthanized. Their heads were removed and then placed in a 10% neutrally buffered formalin solution for fixation. Subsequently, the buccal mucosa and tongue of each rat were decalcified, dehydrated, dealcoholized, and embedded in paraffin for further processing.

Histological Examination

Thin sections of the tissue samples embedded in paraffin were prepared and stained before histological examination under a light microscope [17]. An untreated rat served as the control for comparison purposes.

Ethical Approval

The study protocol, including animal handling and experimentation, was approved by the Ethical Committee of the College of Pharmacy, University of Baghdad, under code number RECAUBCP372023L.

Stability Study of the Optimum FFS Formula

The best fluconazole oral spray solution formula was evaluated by a stability test, by keeping three samples of selected formula in sealed containers at 25°C, 40℃, and 5℃ conditions for three months according to the International Council for Harmonization. The sample F11 was reexamined for pH, drug content, and viscosity [18].

Statistical analysis

One-way analysis of variance (ANOVA) was the method used to conduct the statistical analysis followed by the least square difference post hoc test. If the value of p was equal to or less than 0.05, then the differences were regarded to be statistically significant. The Microsoft Excel program was utilized at every stage of the data analysis process.

## Results

Characterization of fluconazole

Determination of Fluconazole λ Max in Ethanol and Buffer Solution of pH 6.8

**Figure 1:**
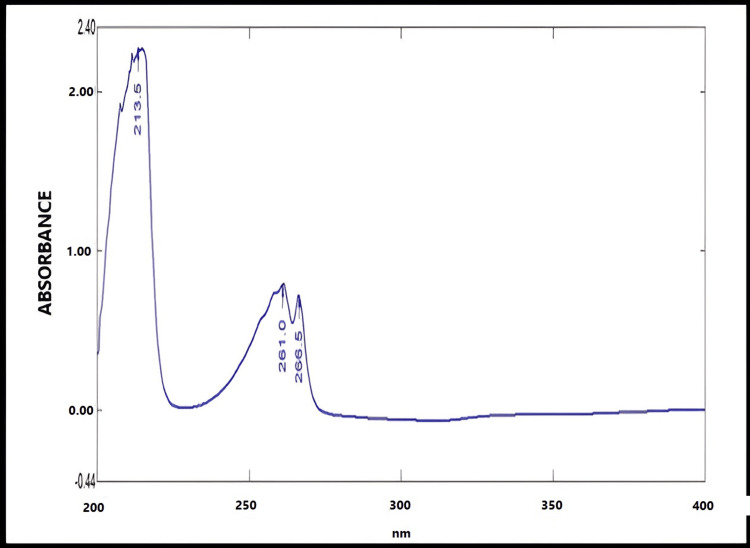
UV spectrum of fluconazole in ethanol

**Figure 2:**
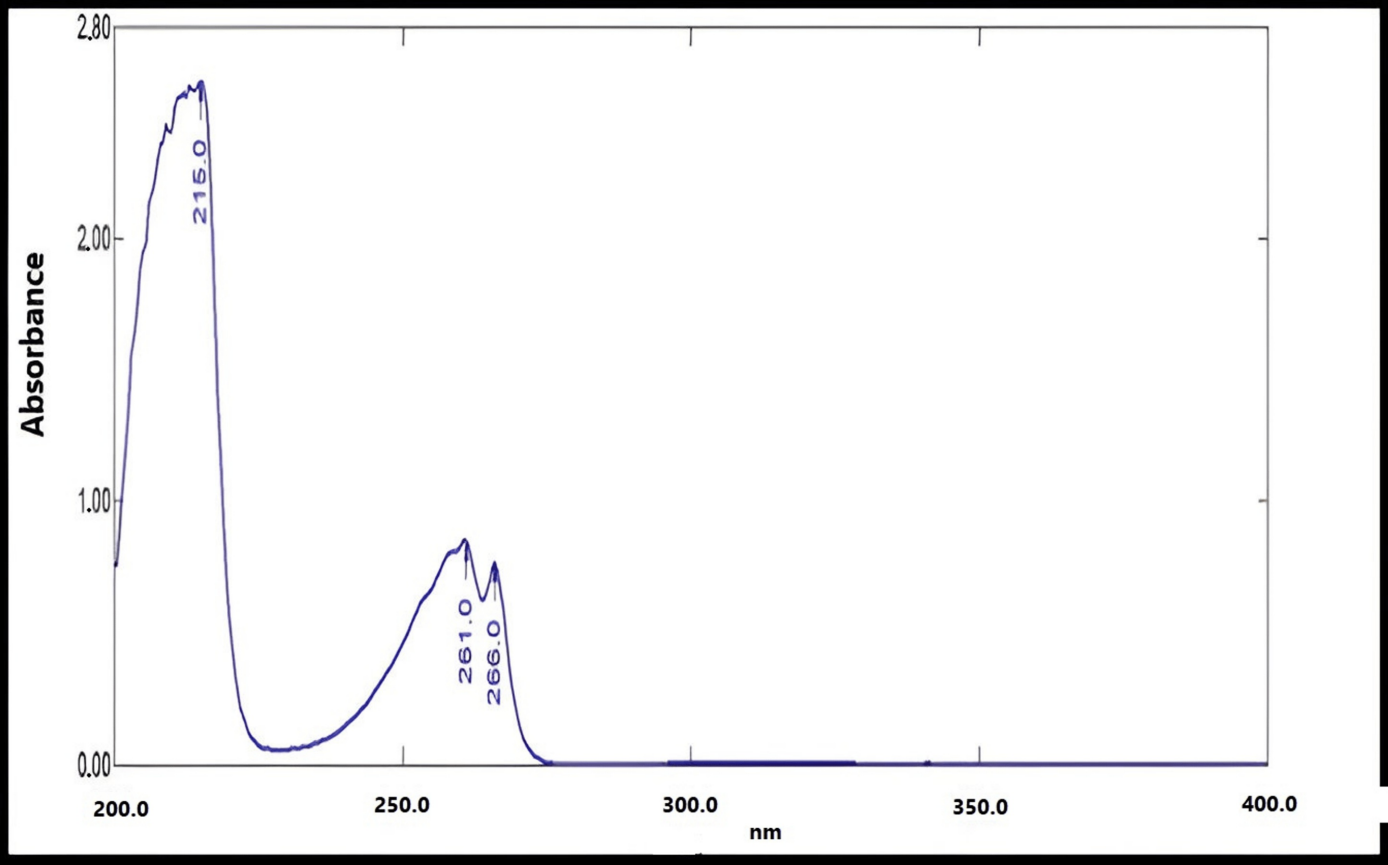
UV spectrum of fluconazole in phosphate buffer of pH 6.8

The obtained UV spectra of fluconazole in ethanol and phosphate buffer of pH 6.8 exhibited maximal peaks at 261 nm. The corresponding results are shown in Figures 1, 2.

Calibration Curves of Fluconazole in Ethanol and Buffer Solution of pH 6.8

**Figure 3:**
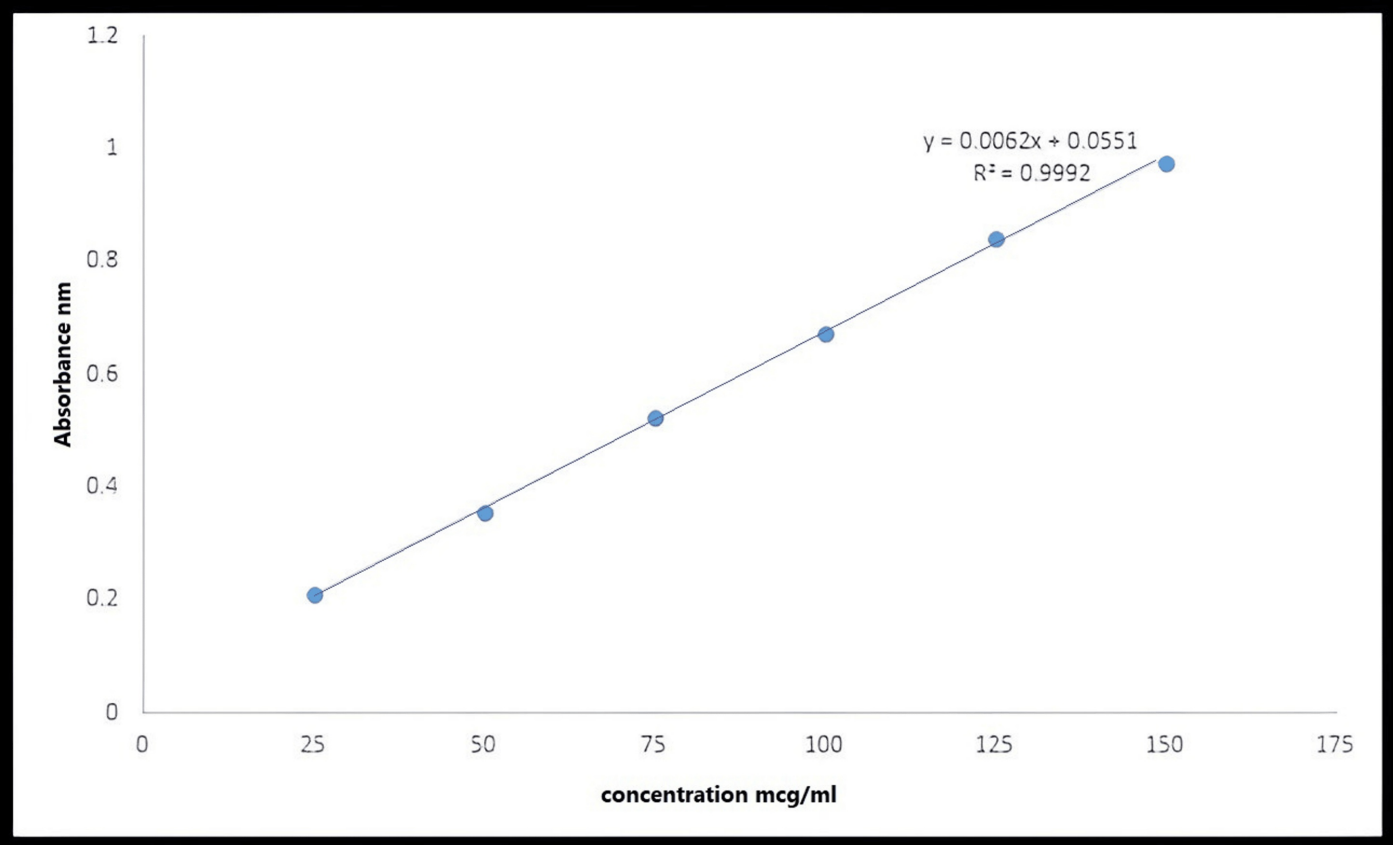
Calibration curve of fluconazole in ethanol

**Figure 4:**
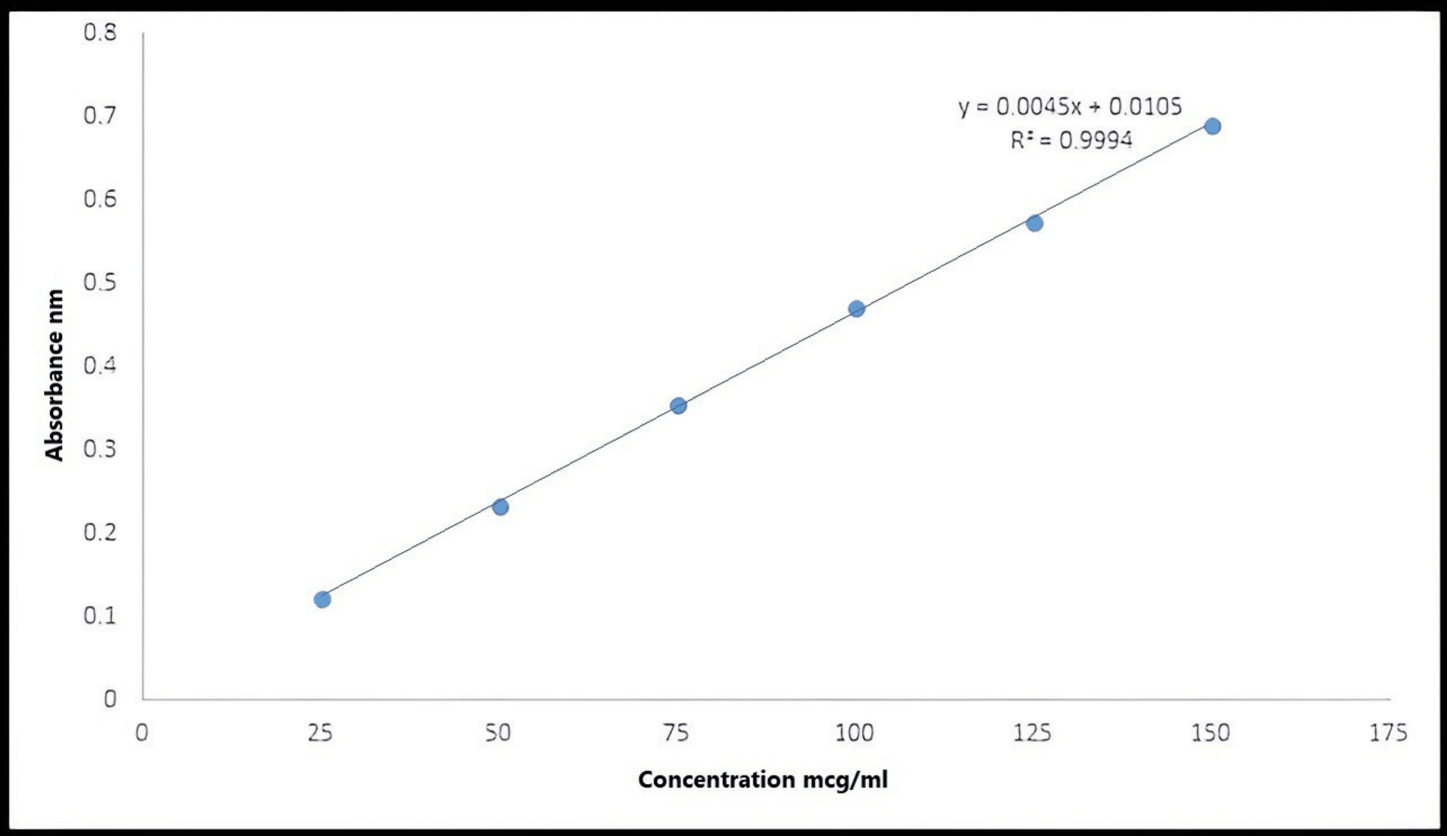
Calibration curve of fluconazole in phosphate buffer of pH 6.8

The fluconazole calibration curves were measured in ethanol and phosphate buffer 6.8 as indicated in Figures 3, 4, respectively. Using Microsoft Excel, all of these curves were created by graphing dilution concentrations in µg/mL against their corresponding absorbance.

FTIR Compatibility Test of Drug and Excipients

**Figure 5:**
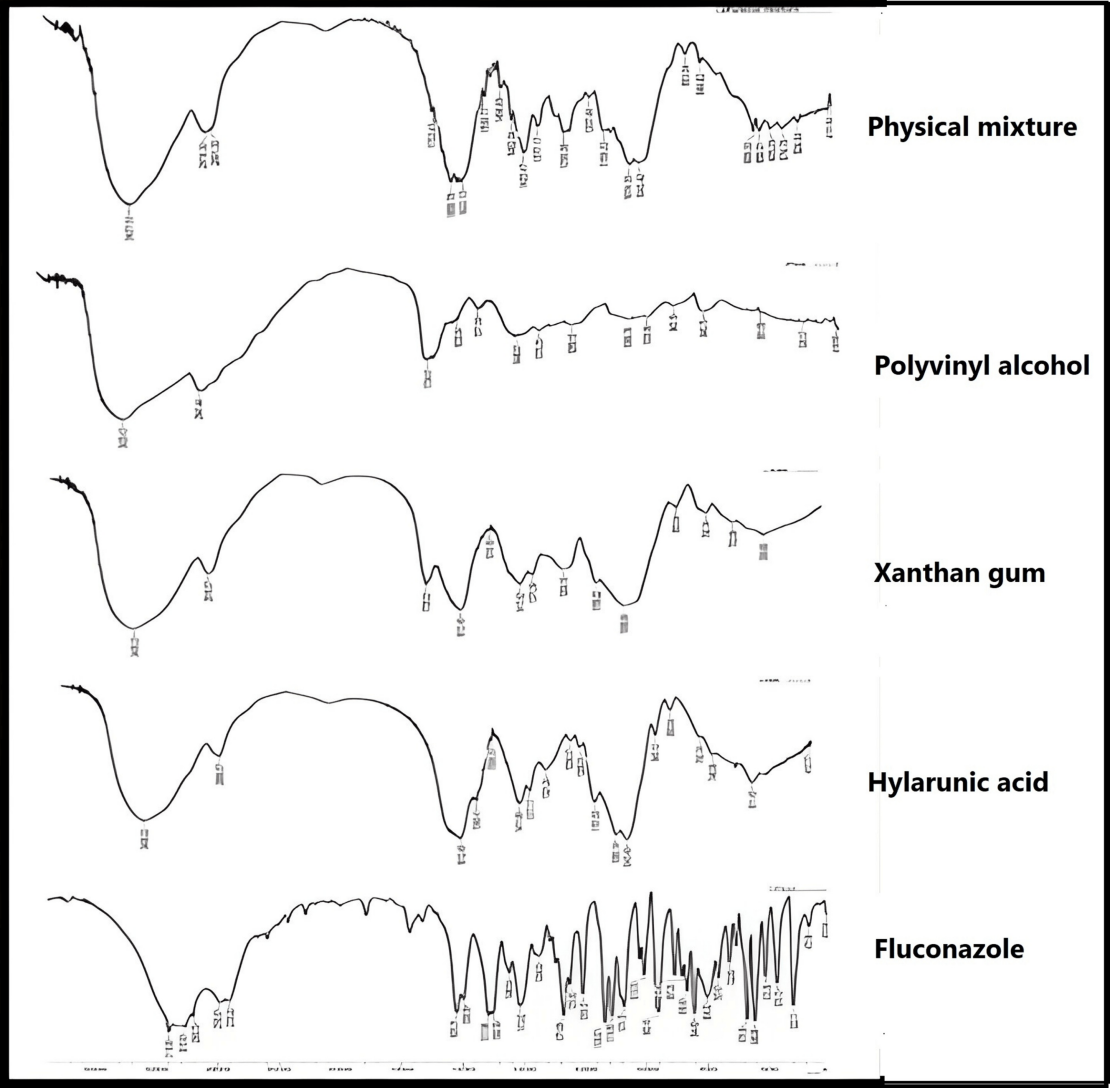
FTIR spectra show the fluconazole, hyaluronic acid, xanthan gum, polyvinyl alcohol, and physical mixture from bottom to top

The FTIR spectra of fluconazole, polymers used in formulas, and the physical mixture of fluconazole and polymers used are shown in Figure 5.

Fluconazole content determination in prepared formulas

**Figure 6:**
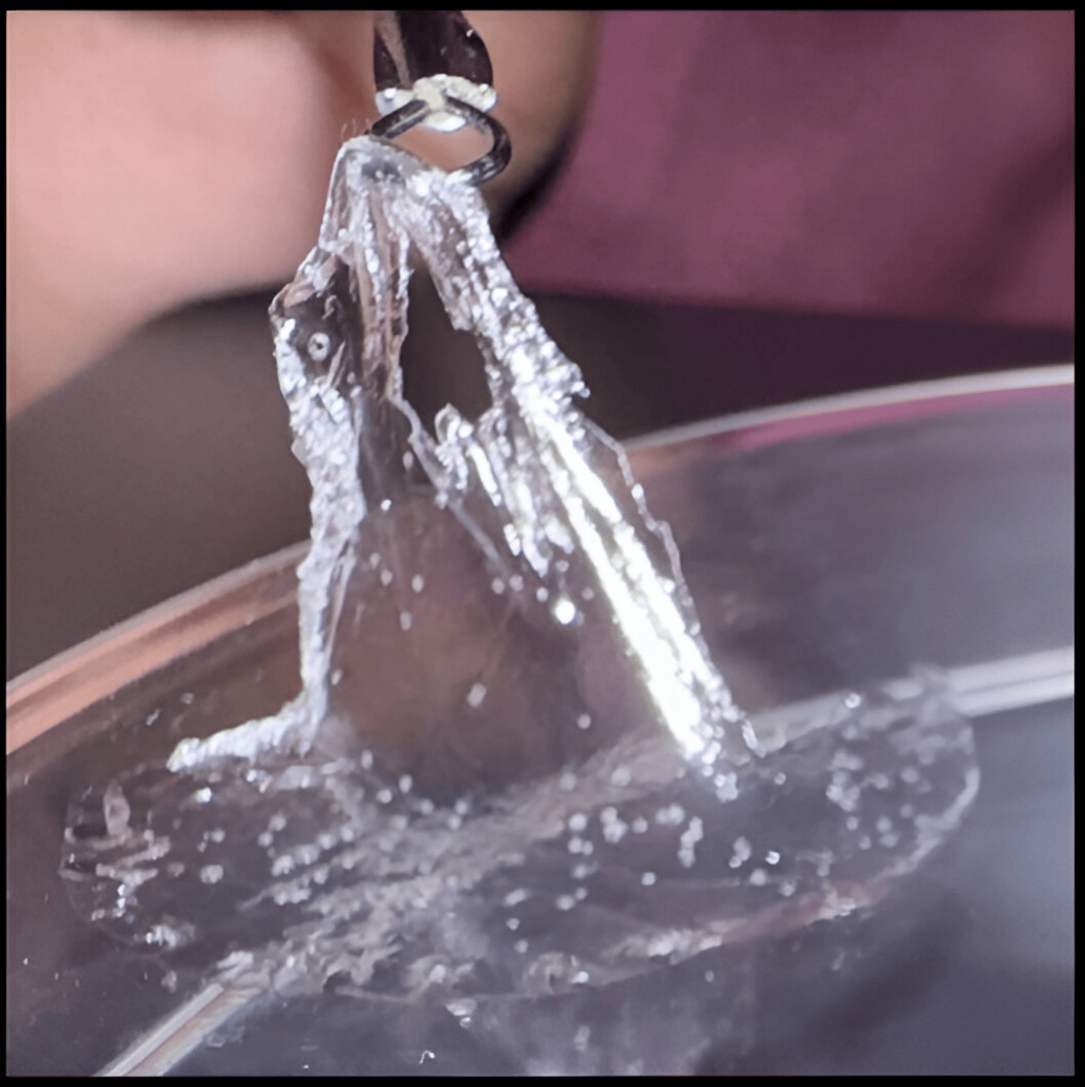
F11 film formation after 55 sec

Table 2 displays the drug content (%) and film drying time for all seven formulas of fluconazole it is observed that fluconazole content for all prepared mucoadhesive formulas is between (88.6%-102%). The F11 formula exhibited the exhibited the optimum values of both fluconazole concentration and film drying time (Figure 6). Time for film formation results indicate the more polymer content contributes with faster drying time.

**Table 2:** Drug content of film-forming solutions and time for solvent evaporation and film formation

Formula	% Drug content	Film drying time (sec.)
F1	104	170
F2	102.1	130
F3	97.3	115
F4	98.2	105
F5	95.6	86
F6	97.8	59
F7	90.6	70
F8	94.9	100
F9	93.5	75
F10	101.1	60
F11	99.7	55
F12	88.6	58
F13	91.1	70

Viscosity Determination

As shown in Table 3, in preparations containing only one type of each polymer (PVA, HA, XG), in the same concentration of (0.3%), at a low speed of 30 rpm, the XG aqueous solution is more viscous than the HA aqueous solution. And both are more viscous than PVA aqueous solutions. But at a higher shear rate of 100 rpm, the XG solution is less viscous than HA. With F11, F12, and F13 containing 10, 20, and 30% PEG400, respectively, increasing PEG400 concentration increased viscosity.

**Table 3:** Viscosity of the prepared oral film-forming solution formulas at different speed (rpm)

Formula	Viscosity at different speed (cp)			
	12 rpm	30 rpm	60 rpm	100 rpm
F1	35±15.2	27±7.7	22±2.2	15±5.2
F2	45±8.1	33±2.5	24±3.5	20±4.0
F3	160±12.0	148±5.2	134±7.5	118±3.9
F4	225±6.8	201±4.9	169±3.0	140±3.7
F5	350±4.4	200±13.2	128 ±9.2	88.8± 4.4
F6	473±5.4	260±6.1	182±13.2	111±2.9
F7	182±9.0	162±19.8	144±6.3	131±3.9
F8	262±7.7	243±6.3	205±8.2	155±4.2
F9	310±11.2	260±6.2	222±4.1	190±5.3
F10	380±4.4	319±5.8	262±8.1	219.9±5.0
F11	1158±6.2	733±4.1	472±5.3	378±3.0
F12	1220±3.5	796±9.2	566±2.2	434.4±3.8
F13	2170±27.2	1244±2.3	878±7.3	690±5.5

Mucoadhesion Strength by Turbidimetric Method

**Figure 7:**
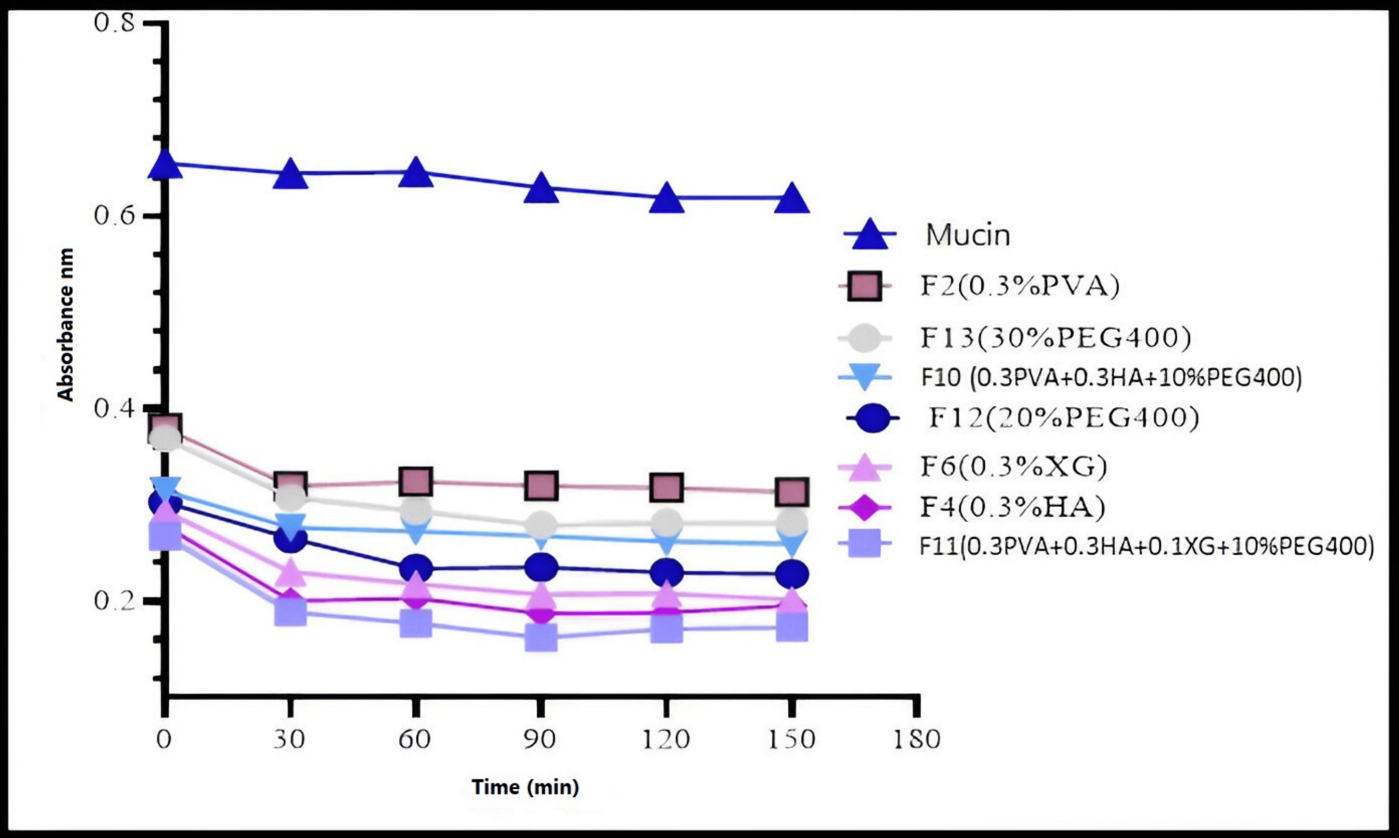
Mucoadhesion strength represents by the absorbance of interaction between prepared formulas and mucin

The absorbance of a mixture of mucin and each formula was compared to that of a dispersion of mucin alone at a wavelength of 650 nm using turbidimetric measurements taken with an ultraviolet-visible spectrophotometer. The absorbance of a 0.1% aqueous mucin dispersion at λ=650 nm did not substantially alter, hence changes in turbidity of formula-mucin dispersions should be seen as an indication of an eventual interaction between the two substances, rather than particles moving around. Turbidity and mucoadhesion are both enhanced when absorbance decreases. As seen in Figure 7, HA solution is more mucoadhesive than XG solution and both are more mucoadhesive than PVA solution. In F13, F12, and F11 increasing PEG400 concentration decrease mucoadhesion. F11 with XG is more viscous than F10 same formula without XG.

Drug Release Study

**Figure 8:**
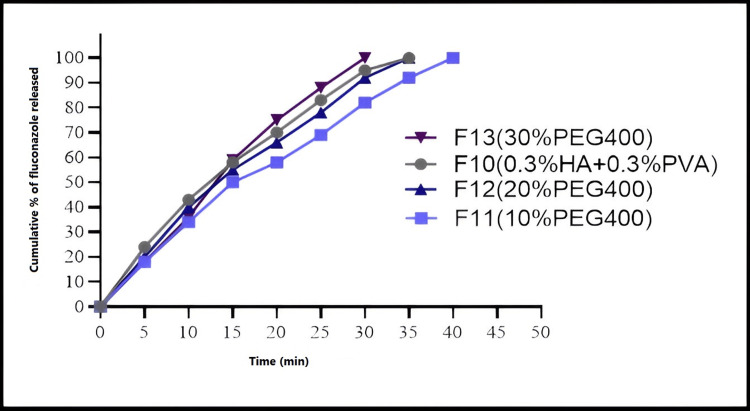
In-vitro fluconazole dissolution from prepared film forming spray in phosphate buffer (pH 6.8) at 37°C

In formulas containing same ingredient with different PEG 400 concentration, the maximum release was seen with F13 film containing 30% PEG400, followed by F12 with 20% PEG400, then F11 with 10% PEG400. F10 and F11, same ingredients but F11 contain XG, release from F10 was faster (Figure 8).

Selection of the optimum mucoadhesive film-forming spray solution formula

F11 was selected as the best formula, as it gave the highest mucoadhesion and very fast drying time.

Evaluation of the optimum formula

In-Vitro Antifungal Activity Study

**Figure 9:**
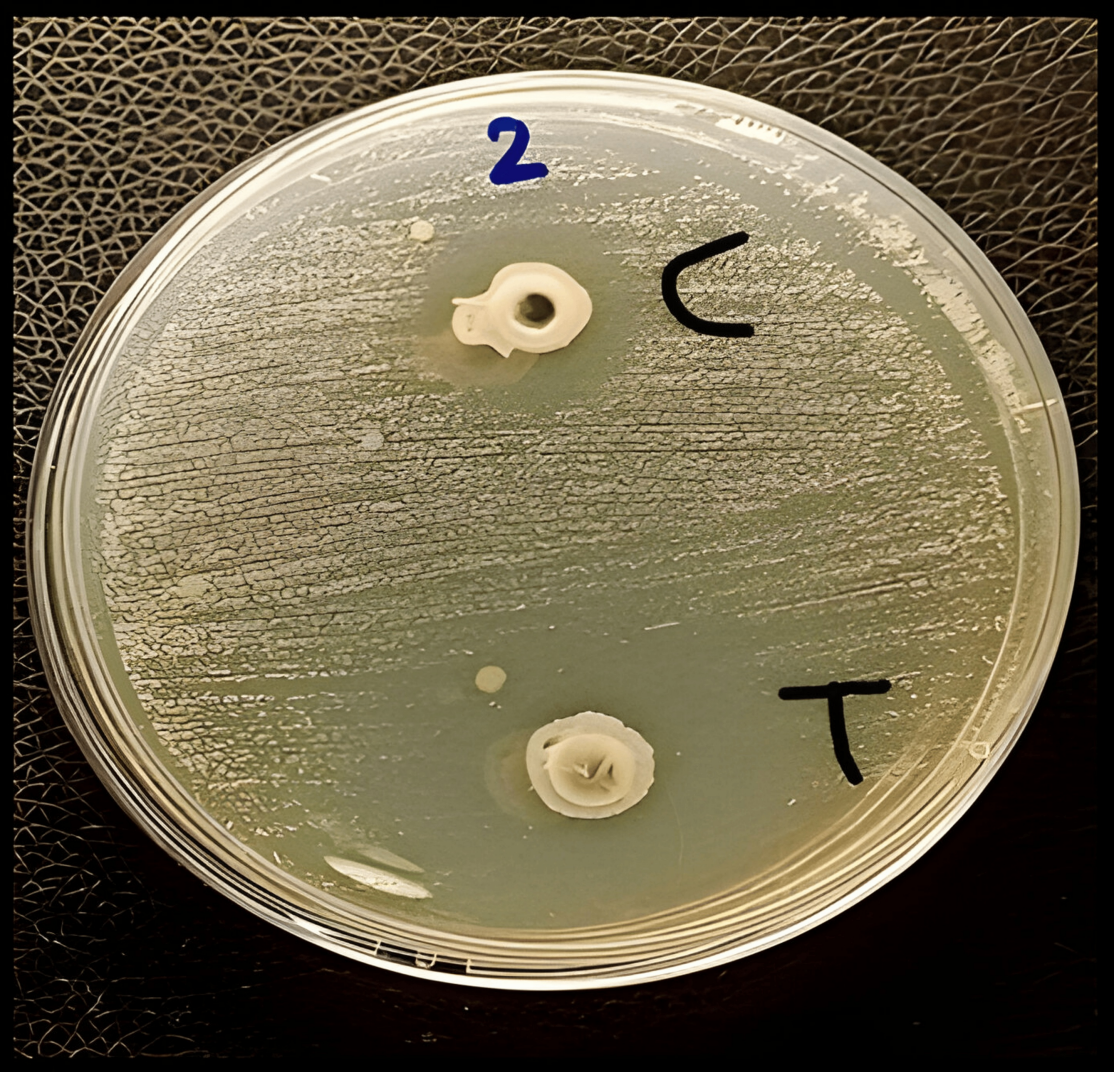
Anti-Candida albicans activity for F11, where is (T) oral spray solution containing fluconazole shows an inhibition zone of 30 mm against C. albicans, (C) control formulation of oral spray without fluconazole shows an inhibition zone of 10 mm

F11 exhibited an inhibition zone of 30 mm against *C. albicans* and an inhibition zone of 10 mm was observed in the control group (without fluconazole) as shown in Figure 9.

Irritancy test for the selected spray formula

Macroscopic Examination

**Figure 10:**
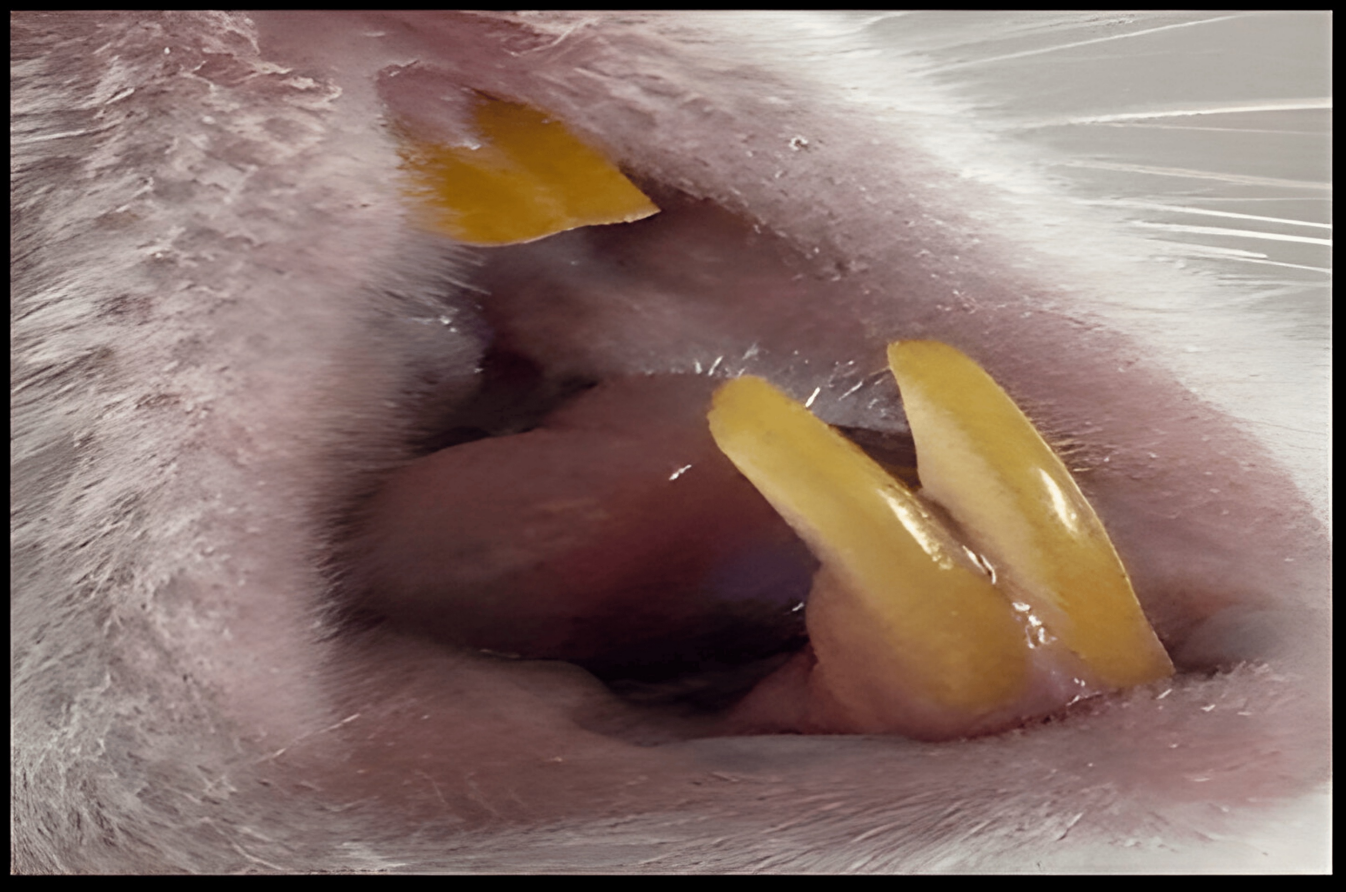
Macroscopic examination of rat after 24 hours from application of F11

The tongue dorsum and buccal mucosal regions were examined before and after F11 application as shown in Figure 10. Oral liquid formula F11 was applied to animals and examined after one, two, and 24 hours without causing any adverse reactions, including discoloration, ulceration, or irritation, at the examination sites.

Histopathological Examination

**Figure 11:**
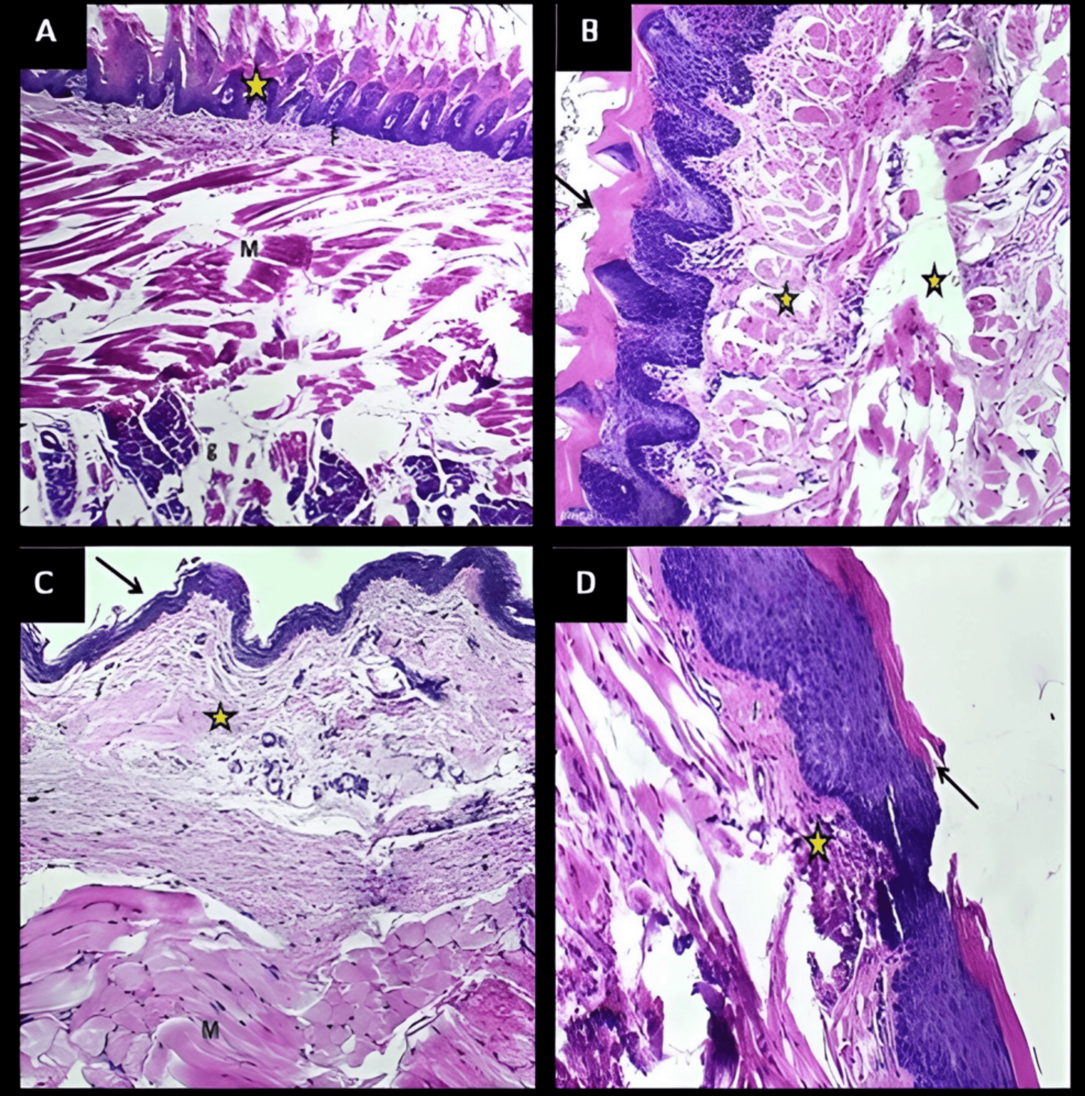
(A) Section of normal (control) lingual mucosa, (B) section of lingual mucosa of rat after 24 from exposed to F11, (C) section of normal buccal mucosa shows normal mucosal with normal lining epithelium (arrow), (D) section of normal buccal mucosa after 24 from exposed to F11 (arrow) Asterisk indicates normal lining epithelium (A) and normal fibrous tissue (B-D)

The histopathological figure of the tongue of treated rat (Figure 11B) revealed normal appearance that is similar control negative (Figure 11A) in which the section of normal lingual mucosa shows: normal mucosal surface with normal lining epithelium (asterisk), normal fibrous tissue (F), lingual muscles (M) and glossal salivary glands (g). While in Figure 11D buccal mucosa for treated rat revealed normal lining epithelium like other figure of buccal mucosa similar that of control group (Figure 11C), in which the section of normal buccal mucosa shows normal mucosal surface with normal lining epithelium (Arrow), normal fibrous tissue (asterisk), and buccal striated muscles (M).

Stability study of the optimum film forming spray formula

There is little to no changes in pH, viscosity, and drug content after keeping the formula F11 at 5°C, 25°C, and 40°C for three months as seen in Table 4.

**Table 4:** Stability parameters of optimum preparation F11 at different temperatures

Parameter	Temperature (°C)		
	5°C	25°C	40°C
pH	5.8	5.9	5.8
Viscosity (CP)	380	377	375
Drug content (%)	99.0	98.3	97.2

## Discussion

FTIR compatibility test of drug and excipients

Figure 5 shows the FTIR spectra for pure fluconazole, which is identical to the reference drug. The FTIR of the physical mixture of drug and polymers was obtained to identify any possible chemical interactions. For pure fluconazole O-H hydrogen bond stretching, observed from 3,200 cm^-1^ to 2,600 cm^-1^, peaks were observed at 1,502 cm^-1^ and 1,414 cm^-1^ due to triazole ring stretching; peak at 1,136 cm^-1^ for triazole ring breathing vibration; and peaks at 962 cm^-1^ and 844 cm^-1^ for C-H bending of triazole ring and 619 cm^-1 ^and 1,514 cm^-1^ for C=C. The physical mixture of fluconazole and polymers exhibits most of the bands associated with the triazole ring, which contributes to the fluconazole's anti-fungal activity and validates the prepared formulas' activity [19].

Viscosity analysis

XG is a polysaccharide, a complex sugar molecule forming hydrogen bonds with water, XG has a high molecular weight and a very long chain-like structure. These long chains become entangled in solution, resisting flow and making the liquid thicker and more viscous [20]. Also, the presence of negatively charged side groups acetate and pyruvate on sugar units results in electrostatic repulsion, leading to the expansion of the chains and thus increased resistance to flow [21]. The linear polysaccharide HA is composed of disaccharide units, glucuronic acid, and N-acetyl glucosamine. It establishes hydrogen bonds between its carboxyl and acetyl groups with water. Due to its high molecular weight and great affinity for water, HA has a high viscosity when dissolved in water [22]. PVA 1788 is a low molecular weight grade of PVA, because it polymerizes to a lesser extent, in comparison with HA it has a lower molecular weight. This indicates that its molecular chains are shorter. Additionally, the reduced frequency of entanglement between shorter chains facilitates their movement in aqueous solutions leading to lower viscosities [23]. Due to the above reasons, at low shear rates of 30 rpm and 60 rpm, the F6 (0.3% XG) aqueous solution is more viscous than the F4 (0.3% HA) aqueous solution. Both are more viscous than F2 (0.3% PVA) aqueous solution. But at higher shear rates 60 rpm and 100 rpm, XG solution is less viscous than HA. The reason is that although both polymers are shear-thinning, XG solutions have strong shear-thinning behavior [24]. HA solutions are shear-thinning non-Newtonian liquids. Due to the hydrophobic effect at higher shear rates and the breakdown of intramolecular hydrogen bonds. Thus, the deformation of HA molecule chains and the flow in the direction reduce the viscosity of the solution [22]. That is what was observed in F4 which increases in shear rate (rpm) and decreases the viscosity. In F2, PVA exhibits non-Newtonian shear-thinning behavior which agrees with the results observed with rheological behaviors of PVA/H_2_O Solutions of High-Polymer Concentration. With F11, F12, and F13 containing 10%, 20%, and 30% PEG, respectively, increasing PEG concentration which is oligomer has relatively low molecular weight and high chain flexibility, however, increases interchain entanglement of polymer chains making it harder for a solution to flow freely, and thus increase viscosity [25]. That was similar to clotrimazole transdermal spray with different concentrations of PEG 400.

Mucoadhesion strength

An efficient buccal mucosal delivery system will keep its tip in close contact with the mucous layer that sits atop the epithelial tissue at all times [26]. Epithelial cells release a protein called mucin, which is the primary component of mucus. The complex process of mucoadhesion begins when mucin and polymer establish force bonds, which may be ionic, hydrogen, covalent, or van der Waals [27]. Because there was no significant difference in the absorbance of a 0.1% aqueous mucin dispersion at λ=650 nm, any changes in turbidity of formula-mucin dispersions conducting the turbidity mucoadhesion test should be taken as an indication of an interaction between the two components, rather than just particles movement.

Xanthan gum, a unique polysaccharide, is known for its mucoadhesive strength [28]. Because of its unique structure. Side chain groups like acetyl and pyruvate can form hydrogen bonds with the mucin, and their high molecular weight allows them to form entanglement with the mucin network within the mucus, resulting in strong mucoadhesion [29]. Despite PVA having more mechanical properties than HA. HA has a greater mucoadhesion strength [7]. As a result, F2 (0.3% HA) has greater muco-adhesion than F4 (0.3%PVA). The reason behind this is that even both polymers are capable of forming hydrogen bonds with mucin, [30]. The improved mucoadhesive properties of HA might be due to two factors: first, HA has viscoelastic behavior similar to that of mucin, providing strong and long mucoadhesion. Second, its ability to undergo swelling and absorb water leads to increased surface contact and interaction with mucosal tissues [31]. As a result, F4 (0.3% HA) is more mucodhesive than F6 (0.3% XG), and F2 (0.3% PVA) has the lowest mucoadhesion strength. F4 (0.3%HA) gave higher mucoadhesion than F10 (0.3%HA+0.3%PVA) the reason might be because PVA creates a physical barrier for mucoadhesion of HA to mucin. However, PVA was added due to its mechanical strength and excellent film-forming film, PVA is biodegradable and capable of decreasing the rate of HA and other natural polymer degradation and erosion rates. The addition of XG in F11 (0.3HA+0.3PVA+0.1XG) exhibits greater mucoadhesive strength compared to F10 (0.3HA+0.3PVA+no XG). This is similar to the result of the combination of HA and XG to make oral mucoadhesive lozenges, which gave a synergistic effect on mucoadhesion duration and strength on mucosal surfaces in an in-vivo study. This indicates that the combination of XG and HA had a synergistic effect on mucoadhesion [10].

Fluconazole release study

Figure 8 showed that fluconazole release from F13 (30%PEG) formula was higher than that from F12 (20%PEG) and both higher than that from F11 (10%PEG). This finding was due to the high solubility of Fluconazole in PEG400 [32]. A similar observation was reported in the release of Fluconazole from Fluconazole ointment containing different amounts of PEG400 [33]. F10 (0.3HA+0.3PVA+no XG) and F11 (0.3HA+ 0.3PVA+ 0.1XG) both contain the same ingredients in the same concentration; with the exception, F11 contains 0.1% XG. F11 contains 0.1% XG, and fluconazole release from F11 was lower than that of F10. XG is known for its drug release-delaying properties due to its gelling properties and ability to entrap drugs in the gel matrix. Drugs are then released through diffusion [34].

The fluconazole optimal formula F11 exhibited an inhibition zone of 30 mm against *C. albicans*, and an inhibition zone of 10 mm was observed in the control group (without drug). This might be due to the anti-candida properties of ethanol.

Irritation study

The histopathological examination of both control (untreated) and treated rats revealed a normal appearance of lingual structure and cytoarchitecture. It showed normal lingual papillae, epithelium, fibrous laminal propria, and normal internic bundles of skeletal muscle of the rat’s tongue. On the other hand, the histopathological figures of the oral mucosa in both control and treated rats also revealed normal appearance structure and cytoarchitecture that composed of normal thin keratinized stratified squamous epithelium that supported by normal thick fibrous lamina propria, adipose tissue and normal bundles of the masseter muscle (Figure 11). Since there was no irritation or harmful response on test animals at the examination locations, F11 is considered safe.

Stability study of the FFS formula

After three months of F11 storage at temperatures of 5°C, 25°C, and 40°C, preparation viscosity, pH, and drug content were determined. The resulting mucoadhesive film-forming oral spray mixture is suitable for storage in the refrigerator and at other temperatures.

Limitations of the study include clinical trials involving human subjects are necessary to validate the effectiveness and safety of the proposed oral spray in real-world scenarios and the need for long-term stability according to ICH.

## Conclusions

The study findings illuminate key insights into the formulation and evaluation of mucoadhesive films for controlled drug delivery. The study underscores the significance of mucoadhesion strength, as evidenced by the absorbance of preparations and mucin interactions. Variations in PEG 400 concentrations influenced drug release profiles, with formulations like F13 exhibiting optimal release characteristics. F11 emerged as the preferred formula due to its exceptional mucoadhesive properties and rapid drying attributes. In vitro assessments demonstrated F11's potent antifungal efficacy, surpassing the control group in inhibiting *C. albicans* growth. F11's safety profile and stability across varying temperatures make it a promising candidate for further development. The combination of XG and HA in F11 showcased enhanced mucoadhesive strength, aligning with previous research on mucosal adhesion. The study advocates for clinical trials to validate the formula's efficacy in real-world scenarios and emphasizes the importance of long-term stability assessments. Collectively, these findings position F11 as a promising contender in the realm of mucoadhesive drug delivery systems, warranting further exploration and validation in clinical settings. The study results demonstrate the success of this research in formulating and refining a mucoadhesive oral spray for treating oral candidiasis.
